# The expression of miR-21, HSP90a and gGASP-1 in serum of patients with lung cancer and their correlation with pathological subtypes

**DOI:** 10.5937/jomb0-48051

**Published:** 2024-06-15

**Authors:** Hongyan Pang, Yange Gong, Yaojie Wang, Lianyong Zhang

**Affiliations:** 1 Cangzhou Central Hospital, Department of Respiratory and Critical Care Medicine, Cangzhou, China

**Keywords:** lung cancer, heat shock protein-90a, miR21, G protein-coupled receptor-associated sorting protein 1, pathological subtypes, rak pluća, protein toplotnog šoka-90a, miR-21, G protein-coupled receptor-associated sorting protein 1, patološki podtipovi

## Abstract

**Background:**

To investigate the expression of miR-21, heat shock protein-90a (HSP90a) and G protein-coupled receptorrelated sorting protein 1(GASP-1) in the serum of lung cancer patients and their correlation with pathological subtypes.

**Methods:**

Eighty patients with lung cancer were included in the lung cancer group from May 2020 to May 2022, and 40 volunteers who underwent physical examination were randomly included in the control group according to the group ratio of 2:1. This ratio balances the need for a sufficiently large experimental group to detect significant effects with the practicality of recruiting a manageable control group. To ensure the validity of our findings, we meticulously calculated the sample size to achieve adequate statistical power, thus enabling us to draw reliable conclusions. Serum miR-21, HSP90a and GASP-1 levels of patients in the two groups were detected. We quantitatively assessed the serum levels of miR-21, HSP90a, and GASP1 in lung cancer patients and healthy volunteers. We employed enzyme-linked immunosorbent assay (ELISA) for HSP90a and GASP-1, and reverse transcription-polymerase chain reaction (RT-PCR) for miR-21, ensuring precise quantification. To explore the correlation between it and pathological subtypes, TNM stage and lymph node metastasis of lung cancer patients. TNM stands for Tumor, Node, and Metastasis. This system is widely used for staging cancer and describes the size and extent of the primary tumor (T), the absence or presence of cancer in nearby lymph nodes (N), and whether the cancer has spread to other parts of the body (M).

**Results:**

The serum levels of miR-21, HSP90a and GASP1 in lung cancer group were higher than those in control group (P < 0.05). ROC curve analysis showed that serum miR-21, HSP90a and GASP-1 levels had certain value in the diagnosis of lung cancer, and their AUC values were 0.901, 0.874 and 0.865, respectively (P < 0.05). There was no difference in the relative expression level of serum miR-21 between squamous cell carcinoma group and adenocarcinoma group (P>0.05), but the levels of HSP90a and GASP-1 in adenocarcinoma group were higher than those in squamous cell carcinoma group (P < 0.05). There was no difference in the levels of serum miR-21, HSP90a and GASP-1 between stage I and stage II groups (P>0.05). The levels of serum miR-21, HSP90a and GASP-1 in stage III and stage IV groups were higher than those in stage I and stage II groups, and those in stage IV were higher than those in stage III group (P < 0.05). The serum levels of miR-21, HSP90a and GASP-1 in patients with metastasis were higher than those in patients without metastasis (P < 0.05).

**Conclusions:**

Our study concludes that there is a notable association between elevated serum levels of miR-21, HSP90a, and GASP-1 and lung cancer. However, it is crucial to acknowledge that these findings are preliminary and further statistical analysis is needed to strengthen these associations. Future studies with comprehensive statistical evaluation will be vital to validate these potential biomarkers for lung cancer diagnosis and prognosis.

## Abstract (sr)

**Uvod:**

Cilj je bio da se istraži miR-21, protein toplotnog šoka-90a (HSP90a) i proteina za sortiranje povezanog sa G proteinom 1 (GASP-1) u serumu pacijenata sa karcinomom pluća i njihova korelacija sa patološkim pod - tipovima.

**Metode:**

Od maja 2020. do maja 2022. godine u grupu za karcinom pluća uključeno je 80 pacijenata sa karcinomom pluća, a 40 dobrovoljaca koji su bili podvrgnuti fizičkom pregledu nasumično je uključeno u kontrolnu grupu prema grupnom odnosu 2:1. Ovaj odnos balansira potrebu za dovoljno velikom eksperimentalnom grupom da otkrije značajne efekte sa praktičnošću regrutovanja kontrolne grupe kojom se može upravljati. Da bismo osigurali validnost naših nalaza, pažljivo smo izračunali veličinu uzorka kako bismo postigli adekvatnu statističku moć, što NAM je omogućilo da izvučemo pouzdane zaključke. Detektovani su nivoi miR-21, HSP90a i GASP-1 u serumu pacijenata u dve grupe. Kvantitativno smo procenili nivoe miR-21, HSP90a i GASP-1 u serumu kod pacijenata sa karcinomom pluća i zdravih dobrovoljaca. Koristili smo enzimski imunosorbentni test (ELISA) za HSP90a i GASP-1 i lančanu reakciju reverzne transkripcije-polimeraze (RT-PCR) za miR-21, obezbeđujući preciznu kvantifikaciju. Istražiti korelaciju između njega i patoloških podtipova, TNM stadijuma i metastaza u limfnim čvorovima pacijenata sa karcinomom pluća. TNM je skraćenica za tumor, čvor i metastaze. Ovaj sistem se široko koristi za stadijum raka i opisuje veličinu i obim primarnog tumora (T), odsustvo ili prisustvo kancera u obližnjim limfnim čvorovima (N) i da li se rak proširio na druge delove tela (M).

**Rezultati:**

Nivoi miR-21, HSP90a i GASP-1 u serumu u grupi karcinoma pluća bili su viši od onih u kontrolnoj grupi (P < 0,05). Analiza ROC krive je pokazala da nivoi miR-21, HSP90a i GASP-1 u serumu imaju određenu vrednost u dijagnozi karcinoma pluća, a njihove AUC vrednosti su bile 0,901, 0,874 i 0,865, respektivno (P < 0,05). Nije bilo razlike u relativnom nivou ekspresije miR-21 u serumu između grupe karcinoma skvamoznih ćelija i grupe adenokarcinoma (P>0,05), ali su nivoi HSP90a i GASP-1 u grupi sa adenokarcinomom bili viši od onih u grupi sa karcinomom skvamoznih ćelija ( P < 0,05). Nije bilo razlike u nivoima serumskih miR-21, HSP90a i GASP-1 između grupa stadijuma I i stadijuma II (P>0,05). Nivoi serumskih miR-21, HSP90a i GASP-1 u grupama stadijuma III i stadijuma IV bili su viši od onih u grupama stadijuma I i stadijuma II, a oni u stadijumu IV su bili viši od onih u grupi stadijuma III (P < 0,05). Nivoi miR-21, HSP90a i GASP-1 u serumu kod pacijenata sa metastazama bili su viši od onih kod pacijenata bez metastaza (P < 0,05).

**Zaključak:**

Naša studija zaključuje da postoji primetna povezanost između povišenih nivoa miR-21, HSP90a i GASP-1 u serumu i raka pluća. Međutim, ključno je priznati da su ovi nalazi preliminarni i da je potrebna dalja statistička analiza da bi se ove asocijacije ojačale. Buduće studije sa sveobuhvatnom statističkom evaluacijom biće od vitalnog značaja za validaciju ovih potencijalnih biomarkera za dijagnozu i prognozu raka pluća.

## Introduction

Lung cancer is a common malignant tumor of the respiratory system in clinical practice, which is prone to brain, liver, and bone metastasis, and has the highest mortality rate among malignant tumors. Approximately 80%–90% of lung cancer patients have non-small cell lung cancer, with squamous cell carcinoma and adenocarcinoma being the most common types [1]. Early diagnosis and treatment can improve patient prognosis and prolong survival time. Currently, the gold standard for pathological classification of lung cancer in clinical practice is pathological tissue examination, which requires tissue sampling and has certain invasiveness, with the results being influenced by the accuracy of sampling [2]. Therefore, the search for laboratory serum diagnostic markers has become a hot topic in current clinical research.

Lung cancer is mainly divided into two types: non-small cell lung cancer (NSCLC) and small cell lung cancer (SCLC). NSCLC is the more prevalent type, encompassing various subtypes such as adenocarcinoma and squamous cell carcinoma. The TNM system is vital for staging lung cancer, assessing the tumor size, lymph node involvement, and metastasis. For molecular aspects, focus on miRNA-21, GASP-1, and HSP-90α. MiRNA-21 is known for its role in cancer progression, influencing cell proliferation and apoptosis. GASP-1 and HSP-90α are also critical in lung cancer, with HSP-90α especially noted for its function in protein folding and cancer cell survival. These molecules can be potential biomarkers for lung cancer and are associated with the disease’s progression and metastasis.

miR-21 is a small molecule RNA that plays an important role in the occurrence and development of malignant tumors [3]. Heat shock protein-90α (HSP-90α) is an ATP-dependent, highly conserved molecular chaperone that is highly expressed in various malignant tumor cells [4]. G protein-coupled receptor-associated sorting protein 1 (GASP-1) is a newly discovered malignant tumor marker that is highly expressed in patients with lung cancer, breast cancer, liver cancer, and other malignant tumors [5]. However, its correlation with pathological classification has not been reported. This study explores the expression of miR-21, HSP-90α, and GASP-1 in the serum of lung cancer patients and their correlation with pathological subtypes.

## Materials and methods

### Baseline information

Eighty patients with lung cancer were included in the lung cancer group, and 40 volunteers who underwent physical examination were randomly included in the control group according to the group ratio of 2:1 from May 2020 to May 2022. This study was conducted in accordance with the ethical standards of the Cangzhou Central Hospital. Ethical approval for this research was granted by the Ethics Committee of Cangzhou Central Hospital, with approval number CCHEC2020-037. All procedures performed in studies involving human participants were in accordance with the ethical standards of the institutional and/or national research committee and with the 1964 Helsinki declaration and its later amendments or comparable ethical standards.

Inclusion criteria: (1) Patients in the lung cancer group who meet the Clinical Diagnosis and Treatment Guidelines for Lung Cance, confirmed by imaging and pathological examination to have non-small cell lung cancer [6]. (2) Patients in the lung cancer group are newly diagnosed and have no previous history of lung cancer. (3) No history of previous radiotherapy or chemotherapy. (4) Clinical data is complete and available for the study. The control group consists of healthy volunteers who underwent health check-ups at our hospital during the same period.

Exclusion criteria: (1) Patients with a history of lung surgery. (2) Patients with malignant tumors in other parts of the body. (3) Patients with autoimmune diseases or infectious diseases.

### Methods

All study subjects underwent a 2 mL blood draw from the cubital vein in the morning on an empty stomach. The blood was centrifuged for 10 minutes at 3000 rpm using the TGL-168 centrifuge from Shanghai Anting Scientific Instrument Factory (Shanghai, China) in an environment at 4°C. The serum was extracted and HSP-90α and GASP-1 were detected using ELISA. The HSP-90α kit was from Shenzhen Jingmei Biotechnology Co., Ltd. (Shenzhen, China), and the GASP-1 kit was from Jianglai Biological Technology Co., Ltd. (Suzhou, China). The enzyme-linked immunosorbent assay instrument used was the MK3 microplate reader from Thermo Fisher Scientific (Waltham, MA, USA).The isolation and quantification of miRNA-21 begin with the collection of appropriate biological samples, such as blood or plasma, which can be used either fresh or stored at low temperatures (like -80°C) for later processing. For the isolation process, commercial RNA extraction kits are commonly employed due to their efficiency and convenience. These kits typically involve cell lysis, binding of RNA to a silica column, washing, and elution. The choice of kit is crucial and should be based on the sample type and the desired RNA yield. Following isolation, RNA quantification is performed, with the Qubit microRNA assay being a preferred method for its accuracy in quantifying low abundance targets. For the specific quantification of miRNA-21, quantitative real-time PCR (qPCR) is the method of choice. This involves reverse transcription of the miRNA into cDNA, followed by amplification using specific primers and probes. The qPCR process requires careful optimization, considering factors such as sample quality, reverse transcription efficiency, and primer specificity. Running reactions in triplicate and including appropriate controls is essential for obtaining accurate and reliable data. Additionally, operational factors like ethanol concentration during RNA isolation and the pH or temperature of the eluent can significantly impact RNA recovery, necessitating careful optimization and standardization of these conditions to ensure consistency and reproducibility of results. This comprehensive approach, encompassing careful sample collection, methodical RNA isolation, precise quantification, and rigorous qPCR, is fundamental to accurately assess miRNA-21 levels in biological samples.

An additional 1 mL venous blood sample was taken, and 200 μL of whole blood was accurately extracted into an RNase-Free centrifuge tube. Equal volumes of lysis solution MZ were added to completely separate the nucleic acid-protein complex. After high-speed centrifugation, chloroform was added, and the supernatant was collected by high-speed centrifugation into a new centrifuge tube. Slowly add 1/3 volume of anhydrous ethanol, mix well and transfer to absorption column miRspin, and spin down to collect the effluent. Slowly add 2/3 volume of anhydrous ethanol and mix well, transfer to absorption column miRelute, and spin down to retain the absorption column miRelute. Proteins MRD and washing solution RW were added, centrifuged, and the waste was discarded. This step was repeated twice, and total RNA was extracted. RNA purity was detected using a UV spectrophotometer and RNA concentration was adjusted before performing RT-PCR. miR-21 was detected using RT-PCR, and the instrument used was the ABI 7500 fluorescence quantitative PCR instrument from the United States. RT-PCR, or Real-Time Polymerase Chain Reaction, is a technique used to amplify and simultaneously quantify a targeted DNA molecule. It enables both detection and quantification of a specific sequence in a DNA sample. The process is often used for measuring the expression of a specific gene. The term »real-time« refers to the fact that the data are collected during the process, allowing immediate analysis of results.

The ABI 7300 Real Time PCR System, created by Applied Biosystems (now part of Thermo Fisher Scientific, located in Foster City, California, USA), is an advanced RT-PCR system. It integrates thermal cycling for DNA amplification with fluorescence detection, allowing for real-time observation of PCR product formation. Key features include a 96 well format, accommodating various sample volumes, and four-color detection, making it versatile for applications such as gene expression analysis and SNP genotyping.

### Observational indicators

This study aims to measure and compare the serum levels of miR-21, HSP90α, and GASP-1 in two distinct groups, evaluating their effectiveness as biomarkers in the diagnosis of lung cancer. The study also involves comparing the serum concentrations of miR-21, HSP90α, and GASP-1 across various subgroups to assess their variability and significance in different stages of lung cancer.

### Statistical analysis

Data processing was performed using Statistic Package for Social Science (SPSS) 23.0 software (IBM, Armonk, NY, USA). Categorical data such as patient gender, smoking history, and alcohol consumption history were expressed as (n/%) and were analyzed using the chi-square test. When comparing more than two groups, ANOVA is often used. It tests the null hypothesis that all groups have the same population mean. The one-way ANOVA is used for comparing more than two groups based on one independent variable, while the two-way ANOVA is used for two independent variables. Continuous data such as serum levels of miR-21, HSP90α, and GASP-1 in patients were represented as mean ± standard deviation and were compared between groups using independent sample t-tests. To test data distribution, several statistical methods are used. The Shapiro-Wilk Test is a popular choice for assessing normality, especially for small sample sizes. It examines if data is drawn from a normally distributed population. The Kolmogorov-Smirnov Test, another nonparametric method, compares a sample’s distribution with a reference distribution, often the normal distribution. The Anderson-Darling Test is similar, emphasizing the tails of the distribution. For a more visual approach, the QQ (Quantile-Quantile) Plot graphically compares the quantiles of the sample data to the quantiles of a theoretical distribution, with a linear pattern indicating similarity. Lastly, the Lilliefors Test, a variant of the Kolmogorov-Smirnov Test, is used when the mean and variance are not known and must be estimated from the data. These tools collectively help in understanding the underlying distribution of the data, crucial for choosing appropriate statistical tests and analyses. The diagnostic value of miR-21, HSP90α, and GASP-1 levels in lung cancer was explored using ROC curves. The significance level was set at P < 0.05. Table 1

**Table 1:** Comparison of General Information Between the Two Groups (x̄±s, n/ (%)).

Group	Gender		BMI<br>(kg/m2)	Smoking history		Age<br>(years)	Drinking history	
	Male	Female		Yes	No		Yes	No
Control group<br>(n=40)	23 (57.50)	17 (42.50)	22.08±1.98	16 (40.00)	24 (60.00)	51.66±6.01	19 (47.50)	21 (52.50)
Lung cancer<br>group (n=80)	45 (56.25)	35 (43.75)	22.13±2.16	31 (38.75)	49 (61.25)	51.20±5.59	37 (46.25)	43 (53.75)
*χ^2^/t*	0.017		-0.123	0.017		0.414	0.017	
*p*	0.896		0.902	0.895		0.679	0.897	

## Results

### Comparison of miR-21, HSP90α, and GASP-1 levels between the control group and the lung cancer group

**Figure 1:**
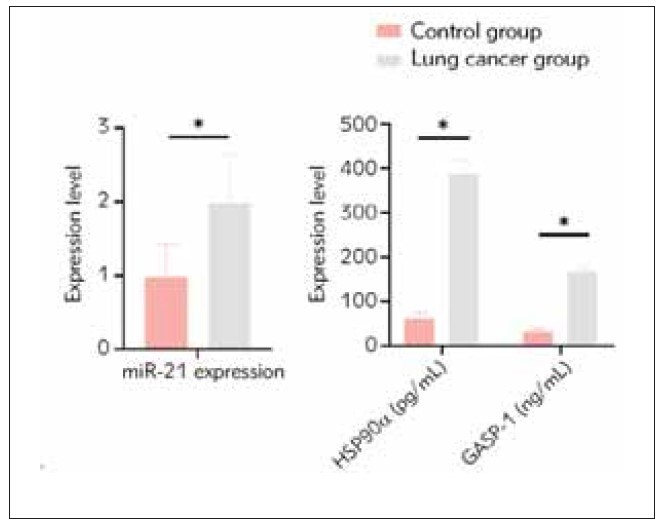
Predictive efficacy of CRP, TNF-α, IL-6, PCT and IL-1β in dexamethasone treatment of refractory purulent meningitis in children.

There were significant differences in serum levels of miR-21, HSP90α, and GASP-1 between the two groups, with higher levels observed in the lung cancer group compared to the control group (Figure 1 and Table 2, P<0.05).

**Table 2:** Comparison of miR-21, HSP90α, and GASP-1 levels between the control group and the lung cancer group (x̄±s). Data compared using t-test.

Group	miR-21<br>expression	HSP90α<br>(pg/mL)	GASP-1<br>(ng/mL)
Control group<br>(n=40)	0.98±0.45	62.33±13.02	31.45±6.45
Lung cancer<br>group<br>(n=80)	1.99±0.65	388.26±29.45	166.29±11.05
*t*	-8.819	-66.703	-71.254
*p*	0.000	0.000	0.000

### The diagnostic value of miR-21, HSP90α, and GASP-1 for lung cancer

**Figure 2:**
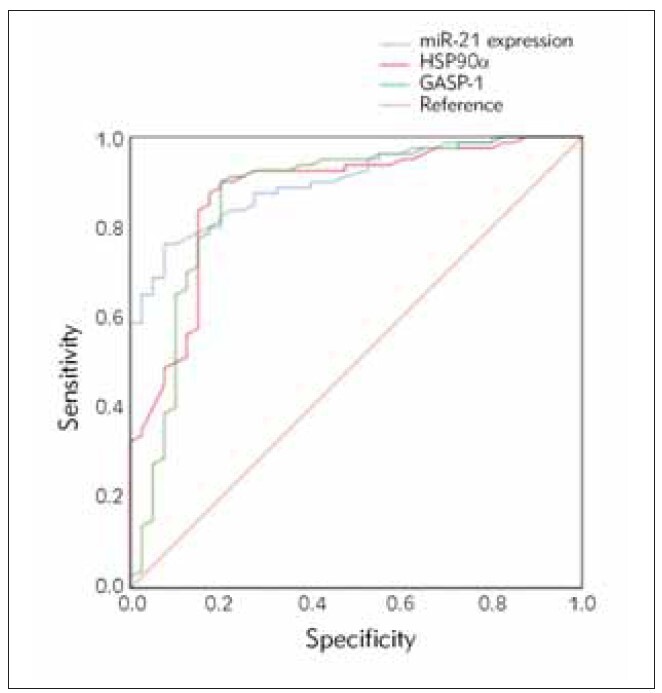
Analysis of the diagnostic value of serum levels of miR-21, HSP90α, and GASP-1 in lung cancer.

According to ROC analysis, miR-21, HSP90α, and GASP-1 have certain diagnostic value for lung cancer, with AUC values of 0.901, 0.874, and 0.865, respectively (Figure 2 and Table 3, P < 0.05).

**Table 3:** The diagnostic value of miR-21, HSP90α, and GASP-1 for lung cancer.

Indicators	AUC	The optimal<br>cutoff value	95%CI		Sensitivity	Specificity	*p*
			Lower	Upper			
miR-21 level	0.901	1.510	0.848	0.953	0.763	0.925	0.000
HSP90α	0.874	197.710	0.805	0.943	0.900	0.800	0.000
GASP-1	0.865	89.360	0.786	0.945	0.879	0.810	0.000

### Comparison of serum levels of miR-21, HSP90α, and GASP-1 between the squamous cell carcinoma group and the adenocarcinoma group

**Figure 3:**
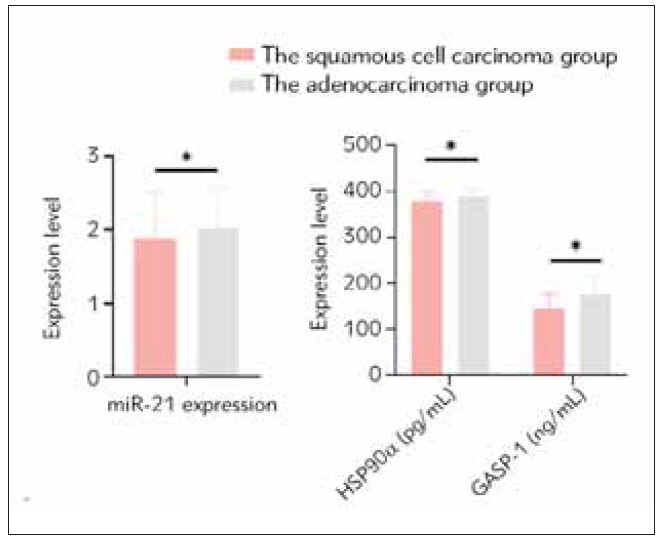
Comparison of serum levels of miR-21, HSP90α, and GASP-1 between the squamous cell carcinoma group and the adenocarcinoma group. *P<0.05.

There was no significant difference in the relative expression levels of serum miR-21 between the squamous cell carcinoma group and the adenocarcinoma group (P > 0.05). However, the levels of HSP90α and GASP-1 in the adenocarcinoma group were higher than those in the squamous cell carcinoma group (P < 0.05). Detailed information was shown in Figure 3 and Table 4.

**Table 4:** Comparison of serum levels of miR-21, HSP90α, and GASP-1 between the squamous cell carcinoma group and the adenocarcinoma group (x̄±s). Data compared using t-test.

Group	miR-21 level	HSP90α (pg/mL)	GASP-1 (ng/m)
The squamous cell carcinoma group<br>(n=31)	1.89±0.64	376.26±23.65	144.26±33.16
The adenocarcinoma group (n=49)	2.02±0.56	387.26±19.26	175.26±40.25
*t*	-1.142	-2.729	-4.207
*p*	0.256	0.007	0.000

### Comparison of serum levels of miR-21, HSP90α, and GASP-1 among different TNM stages.

**Figure 4:**
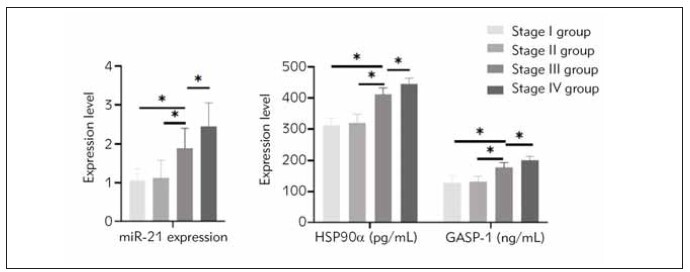
Comparison of serum levels of miR-21, HSP90a, and GASP-1 among different TNM stages. *P<0.05.

There was no significant difference in the levels of miR-21, HSP90a, and GASP-1 between patients with TNM stage I and II (P > 0.05). However, the levels of miR-21, HSP90a, and GASP-1 in patients with TNM stage III and IV were higher than those in the stage I and II groups, and the level in the stage IV group was higher than that in the stage III group (P < 0.05). Detailed information was shown in Figure 4 and Table 5.

**Table 5:** Comparison of serum levels of miR-21, HSP90α, and GASP-1 among different TNM stages (x̄±s). * Indicates P < 0.05 compared with stage I; + indicates P < 0.05 compared with stage II; - indicates P < 0.05 compared with stage III.

Group	miR-21 level	HSP90α (pg/mL)	GASP-1 (ng/mL)
Stage I group (n=12)	1.05±0.31	311.16±24.10	128.33±23.02
Stage II group (n=22)	1.12±0.45	319.15±28.15	131.28±18.26
Stage III group (n=25)	1.89±0.51*+	411.26±21.56*+	177.26±16.26*+
Stage IV group (n=21)	2.43±0.61*+-	445.21±18.59*+-	201.56±12.06*+-

### Comparison of serum levels of miR-21, HSP90α, and GASP-1 between the metastasis group and the non-metastasis group.

Serum levels of miR-21, HSP90α, and GASP-1 were found to be higher in the metastasis group compared to the non-metastasis group (Table 6, P < 0.05).

**Table 6:** Comparison of serum levels of miR-21, HSP90α, and GASP-1 between the metastasis group and the non-metastasis group (x̄±s). Data compared using t-test.

Group	miR-21 level	HSP90α (pg/mL)	GASP-1 (ng/mL)
The metastasis group(n=40)	2.34±0.51	388.15±15.26	188.66±15.23
The non-metastasis group	1.33±0.47	367.10±13.59	144.26±16.59
*t*	10.785	7.675	14.377
*p*	0.000	0.000	0.000

## Discussion

Epidemiological surveys have revealed that lung cancer has the highest incidence and mortality rates, making it the most common malignant tumor in China, with a male-to-female ratio of approximately 1.61:1 [7]. The causes of lung cancer are multifactorial and include smoking, occupational exposure, air pollution, ionizing radiation, malnutrition, and genetic susceptibility [8]. Due to the lack of specific symptoms, a vast majority of lung cancer patients are diagnosed in the middle and late stages, resulting in shorter survival time [9]. Non-small cell lung cancer is the most prevalent type of lung cancer, which includes adenocarcinoma, squamous cell carcinoma, large cell carcinoma, and others, with squamous cell carcinoma and adenocarcinoma being the most common subtypes. Squamous cell carcinoma, accounting for 40–50% of lung cancer cases, has a slow growth rate and a later stage of metastasis but can be treated with surgical resection of the lesion. Adenocarcinoma, accounting for approximately 25% of lung cancer cases, is highly infiltrative and has a higher risk of local and distant metastasis owing to its rich vascularity [10] [11]. Currently, pathological examination is the gold standard for diagnosing lung cancer, but puncture biopsy, an invasive operation is not feasible in patients with contraindications, and puncture sampling may yield inaccuracies [12]. On the other hand, serum examination offers the advantages of easy sampling and almost non-invasive nature, and it is emerging as a new approach in the study of pathological classification and prediction of lung cancer [13].

MiRNA-21 is extensively studied and has been implicated in various types of cancer, including non-small cell lung cancer (NSCLC). It functions as an oncogene and is known for its upregulated expression in lung cancer patients. MiRNA-21 plays a crucial role in the tumorigenesis process by regulating key cellular processes such as the cell cycle, metastasis, angiogenesis, metabolism, and apoptosis. It primarily regulates gene expression through post-transcriptional regulation of mRNA. Interestingly, miRNA-21 is also involved in activating gene expression in some contexts. Its overexpression is closely associated with the progression of lung cancer. For instance, high miR-21 expression has been observed in both cancer cells and cancer-associated fibroblasts (CAFs) in lung adenocarcinoma, which supports tumor progression. The expression of miR-21 in CAFs, particularly in invasive areas of lung adenocarcinomas, is correlated with poor prognosis. This suggests that miR-21 not only plays a role in the growth and spread of lung cancer cells but also contributes to the tumor microenvironment that supports cancer progression. Furthermore, miRNA-21 has been implicated in the regulation of immune checkpoints like PD-1 and its ligand PD-L1, which are crucial for tumor immune escape and creating an environment that favors tumor growth and progression. Understanding the expression and mechanisms of miRNA-21 in lung cancer is essential for developing anti-cancer strategies, including anti-miRs, miR mimics, and microRNA sponges.

This study discovered that the levels of serum miR-21, HSP-90α, and GASP-1 in lung cancer patients were elevated compared to those in the control group. ROC curve analysis indicated that the levels of serum miR-21, HSP-90α, and GASP-1 had significant diagnostic value for lung cancer, with respective AUC values of 0.901, 0.874, and 0.865. These findings suggest that miR-21, HSP-90α, and GASP-1 are highly expressed in the serum of lung cancer patients and can serve as supplementary diagnostic indicators for lung cancer. miR-21, the first serum miRNA discovered, is highly expressed in various malignant tumor cells, but its precise mechanism remains incompletely understood. Previous studies have suggested its involvement in the activation of oncogenes or the inhibition of tumor suppressor genes, with confirmed diagnostic and prognostic value in malignant tumors such as diffuse large B-cell lymphoma, cervical cancer, colorectal cancer, and lung cancer [14] [15]. HSP-90α, a member of the heat shock protein family, is highly expressed under conditions of hypoxia and oxidative stress. It interacts with protein kinases-B, hypoxia-inducible factors, human epidermal growth factor receptor, and others, participating in cell cycle regulation, signal transduction, plasminogen activator activation, and stimulation of neovascularization to promote tumor cell metastasis and infiltration [16] [17]. A study found that serum HSP-90α has good early predictive value in breast cancer [18]. GASP-1 is a recently discovered tumor marker, highly expressed in patients with various malignant tumors [19]. Studies have revealed that the GASP-1 gene can promote microtubule fomationand epithelial-mesenchymal transition in lung cancer A549 cells, facilitating cancer cell invasion and metastasis [20] [21].

This study compared the serum indicators of lung cancer patients with different pathological types. The findings revealed no significant difference in the relative expression levels of serum miR-21 between the squamous cell carcinoma group and the adenocarcinoma group. However, the levels of HSP-90α and GASP-1 in the serum of patients with adenocarcinoma were found to be higher compared to those in the squamous cell carcinoma group. These results indicate that HSP-90α and GASP-1 are linked to the pathological classification of lung cancer, with higher levels being observed in patients with adenocarcinoma. One possible explanation is that adenocarcinoma lesions contain a rich blood supply, and elevated levels of HSP-90α contribute to the stabilization of oncogenic proteins, thereby promoting the proliferation of malignant tumor cells. Moreover, higher levels of GASP-1 can accelerate the formation of microtubules in tumor cells, enhancing their ability for local infiltration and distant metastasis.

This study revealed that there were no significant differences in the serum levels of miR-21, HSP- 90α, and GASP-1 between stage I and II groups. However, higher serum levels of miR-21, HSP-90α, and GASP-1 were observed in stage III and IV groups compared to stage I and II groups, with the highest levels found in stage IV. Furthermore, patients with metastases had elevated serum levels of miR-21, HSP-90α, and GASP-1 compared to those without metastases. These findings indicate that patients with advanced stages of lung cancer and lymph node metastases demonstrate increased serum levels of miR-21, HSP-90α, and GASP-1. Notably, miR-21, HSP-90α, and GASP-1 play a role in activating mutant oncogenic proteins, shielding them from degradation, promoting microtubule formation, and inducing epithelial-mesenchymal transition, ultimately fostering the proliferation of malignant tumor cells. Accordingly, patients displaying higher levels of miR-21, HSP-90α, and GASP-1 tend to exhibit a more advanced clinical staging and a heightened risk of metastasis.

In summary, the serum levels of miR-21, HSP90α, and GASP-1 are elevated in patients with lung cancer, and the levels are associated with the pathological subtypes, staging, and lymph node metastasis of the patients. Therefore, they hold high diagnostic value.

## Dodatak

### Funding

The study was funded by Cangzhou Key R&D Plan Self-raised Project [No: 204106087].

### Conflict of interest statement

All the authors declare that they have no conflict of interest in this work.
